# A cellulolytic fungal biofilm enhances the consolidated bioconversion of cellulose to short chain fatty acids by the rumen microbiome

**DOI:** 10.1007/s00253-019-09706-1

**Published:** 2019-03-07

**Authors:** Charilaos Xiros, Robert Lawrence Shahab, Michael Hans-Peter Studer

**Affiliations:** 1 Laboratory of Biofuels and Biochemicals, School of Agricultural, Forest and Food Sciences, Bern University of Applied Sciences (BFH), CH-3052 Zollikofen, Switzerland; 2 Present Address: RISE Processum AB, SE-891 22 Örnsköldsvik, Sweden; 3 Laboratory of Sustainable and Catalytic Processing, Institute of Chemical Sciences and Engineering, École Polytechnique Fédérale de Lausanne (EPFL), CH-1015 Lausanne, Switzerland

**Keywords:** Biofilms, Membrane reactors, Acetic acid, Butyric acid, *Trichoderma reesei*, *Coprinopsis cinerea*

## Abstract

The ability of the multispecies biofilm membrane reactors (MBM reactors) to provide distinguished niches for aerobic and anaerobic microbes at the same time was used for the investigation of the consolidated bioprocessing of cellulose to short chain fatty acids (SCFAs). A consortium based consolidated bioprocess (CBP) was designed. The rumen microbiome was used as the converting microbial consortium, co-cultivated with selected individual aerobic fungi which formed a biofilm on the tubular membrane flushed with oxygen. The beneficial effect of the fungal biofilm on the process yields and productivities was attributed to the enhanced cellulolytic activities compared with those achieved by the rumen microbiome alone. At 30 °C, the MBM system with *Trichoderma reesei* biofilm reached a concentration 39% higher (7.3 g/L SCFAs), than the rumen microbiome alone (5.1 g/L) using 15 g/L crystalline cellulose as the substrate. Fermentation temperature was crucial especially for the composition of the short chain fatty acids produced. The temperature increase resulted in shorter fatty acids produced. While a mixture of acetic, propionic, butyric, and caproic acids was produced at 30 °C with *Trichoderma reesei* biofilm, butyric and caproic acids were not detected during the fermentations at 37.5 °C carried out with *Coprinopsis cinerea* as the biofilm forming fungus. Apart from the presence of the fungal biofilm, no parameter studied had a significant impact on the total yield of organic acids produced, which reached 0.47 g of total SCFAs per g of cellulose (at 30 °C and at pH 6, with rumen inoculum to total volume ratio equal to 0.372).

**Electronic supplementary material:**

The online version of this article (10.1007/s00253-019-09706-1) contains supplementary material, which is available to authorized users.

## Introduction

The broad and efficient use of plant biomass as a sustainable resource for platform chemicals, fuels, and high added value products is a prerequisite for a bio based circular economy. Within a biorefinery concept, as many biomass components as possible should be valorized in a cost-effective way, targeting a cascade production of different final products. To date, the best-explored and developed biomass biochemical conversion platform is the sugar platform, where the sugars released from the hydrolysis of cellulose and hemicellulose are the intermediates to be fermented to different products (Xiros et al. 2016). A second platform for biomass exploitation is the syngas platform, in which thermochemical systems convert biomass into syngas (mixture of CO, H_2_, and CO_2_) as the feedstock to be further converted to the desired products (Munasinghe and Khanal 2010). A third platform is the carboxylate platform in which biomass feedstocks are converted to short chain fatty acids (SCFAs, organic acids with two to six carbon atoms) as intermediate platform chemicals, using hydrolysis and fermentation with defined or undefined mixed microbial cultures in engineered systems under anaerobic conditions (Holtzapple et al. 1999; Shahab et al. 2018).

SCFAs not only can be sold as commodity chemicals (e.g., acetic acid) or as specialty chemicals (e.g., caproic acid), but they can also be used as platform chemicals. They are important intermediates as they can be converted to many different final products through a large variety of biological or chemical routes such as alcohols (Holtzapple et al. 1999), lipids, polyhydroxyalkanoates, hydrogen (Singhania et al. 2013), fatty acid methyl esters (Jung et al. 2016), and hydrocarbons (Herman and Zhang 2016).

Different approaches have been tried for the biotechnological production of SCFAs, either using specific microbial strains or mixed undefined consortia (Holtzapple et al. 1999; Luo et al. 2018). Since the recovery and separation of SCFAs are one of the costliest steps of the production process (Singhania et al. 2013), the production strategy to be adopted depends mainly on the desired final product and the selectivity required during the fermentation process regarding the SCFAs produced. Although the use of mixed undefined microbial consortia normally leads to a mixture of SCFAs, the fermentation conditions can dramatically affect the composition of SCFAs produced providing the experimenter with valuable tools to tune the process (Ai et al. 2014).

Various natural consortia originating from different sources (sea and lake sediments, anaerobic sludge, compost piles, rumen fluid) have been used for SCFA production (Agler et al. 2011; Dahiya et al. 2015). The use of the rumen microbial community is based on the natural adaptation of this microbiome to the conversion of lignocellulosic substrates to SCFAs, since organic acids constitute the main energy source for the cow metabolism, while methane is a by-product of the rumen metabolism. In *in vitro* studies, the metabolism of the existing methanogenic population has to be inhibited by the addition of chemicals (Chan and Holtzapple 2003; Zhou et al. 2011; Yin et al. 2016) in order to prevent the loss of SCFAs in the form of methane. Mainly acetic and propionic and at a lesser extent butyric acid are in most cases the SCFAs produced in the cow rumen. The pH of the rumen is usually above 5.9 and below 6.5, while the temperature in the rumen ranges from 38 to 40 °C (Weimer et al. 2009). During *in vitro* experiments, at different conditions (e.g., temperature, pH), microbial communities use different metabolic pathways to digest biomass, shifting the compositions and impacting the yields of acids produced (Fu and Holtzapple 2011).

In this study, the microbial community of the cow rumen was studied regarding the production of SCFAs in the multispecies biofilm membrane (MBM) reactors (Brethauer and Studer 2014) using cellulose as the substrate. These reactors are designed for consortium-based consolidate bioprocessing (Shahab et al. 2018). Of course, the conversion of fibers to SCFAs in the rumen is already a “consortium based consolidated bioprocess”, by a microbial community naturally adapted to a cellulosic diet. The existence of both cellulolytic and fermenting microbial strains in the rumen is not only reflected in the physiology of the cow, but is also proved by various studies on the composition of the microbial populations in the rumen (Weimer et al. 2015; Lengowski et al. 2016; Deusch et al. 2017). The MBM system provides ecological niches for aerobic, facultative anaerobic, and obligate anaerobic microbes at the same time allowing the cultivation of the rumen consortium together with a selected aerobic fungus. In the present study, this co-existence aims at enhanced cellulolytic enzyme production during the fermentation, resulting in higher SCFA titers and productivities. Two different fungi were tried as the biofilm forming microorganisms and the effects of various process parameters on SCFAs yield, productivity, and selectivity were investigated.

## Materials and methods

### Individual microorganisms

The fungus *Trichoderma reesei* RUT C-30 (D-86271) purchased from the VTT collection and *Coprinopsis cinerea* (CBS 338.69) obtained from the CBS collection were used in the study. The stock cultures (PDA slants) were kept at 4 °C and were renewed every 3 months.

### Handling of rumen fluid

The rumen microbial community was provided by the Veterinary Department of University of Bern. Rumen fluid was withdrawn form a fistulized cow and transported in the laboratory in serum bottles at controlled temperature. The rumen inoculum from the fistulized cow was withdrawn and acquired in the morning, one and half hours before each experiment to avoid changes due to storage (Granja-Salcedo et al. 2017) The rumen fluid was transported in the lab in closed plastic serum bottles. Handling and filtering of rumen fluid in the lab were done in an anaerobic chamber (LABstar, MBraun, Garching, Germany) at 39 °C using N_2_ as the protection gas. The rumen fluid was filtered through a double cheese cloth before inoculation of the reactors. Fluid of 250 mL was transferred in each reactor. To investigate the effect of inoculum volume on the fermentations, different volumes of rumen fluid (500 mL, 750 mL, and 1000 mL) were centrifuged at 10,000*g*, at 39 °C for 15 min, and the pellets were suspended back to 250 mL of rumen fluid supernatant. For the lowest ratio used, 250 mL of fluid was used as such. Therefore, the volume of the inoculum was kept constant, but not the corresponding amount of microbes in each case. The inoculation of the reactors was done aseptically under N_2_ blanket.

### Diet of fistulized cow throughout the experiments

The experiments were carried out form March 2017 to March 2018. During this period, the fistulized cow was fed differently regarding the amounts of hay and grain/concentrate in its diet (Table S1). The cow calved on the the 7th of June. From 7th of April and on, it received no concentrates at all and the normal hay was changed to a hay made of older grass with a higher fiber content. From 15th of May and on, the hay was changed again to «normal» hay and a small amount of concentrates (whole plant maize cubes, protein and energy concentrates, oligomineral) as well as an oligomineral-bolus were added to the diet. The portion of concentrates was increased on a weekly basis until the peak lactation in September and it was decreased again from January, until March.

### Chemicals and media

All chemicals used in the cultivation media were purchased from Sigma-Aldrich and VWR and were of analytical grade. Cultivations were done in Mandels medium (Xiros and Studer 2017).

### Cultivations in MBM reactors

All fermentations were performed in stirred tank reactors (Labfors 5 BioEtOH, Infors HT, Bottmingen, Switzerland) which were modified in house to MBM reactors as described by Shahab et al. (2018). Briefly, the aeration of the reactors was done with a tubular polydimethylsiloxane (PDMS) membrane (Mono-Lumen Tubing, ID 0.64 x OD 1.19; 50VMQ Q7-4750, Dow Corning, Midland, MI, USA) which was mounted on a stainless steel frame incorporated in the reactor. The air flow through the membrane was 370 N mL min^−1^. The working volume was 2.7 L and the substrate loading (Avicel®, from Sigma-Aldrich, Switzerland) was 1.5% *w*/*v*. The temperature and pH were set and controlled during all experiments. Fermentations started with fungus inoculation, while 48 h afterwards, the rumen consortium was added under anaerobic conditions. As described by Shahab et al. (2018), the gradient of oxygen concentration in the MBM system led to a fungal biofilm formation on the membrane which acted as an oxygen sink. This resulted in anaerobic conditions in the broth at the time of rumen consortium addition. The redox balance was monitored throughout the experiments using redox probes (EasyFerm Plus ORP Arc, Hamilton, Bonaduz, Switzerland). The medium used was described in detail earlier (Xiros and Studer 2017).

### Analysis of metabolites

SCFAs, glucose, and cellobiose were quantified by high-performance liquid chromatography (Waters 2695 Separation Module, Waters Corporation, Milford, MA, USA) as previously described (Shahab et al. 2018) using an Aminex HPX-87H column (Bio-Rad, Hercules, CA, USA) at 65 °C. The mobile phase was 5 mM H_2_SO_4_ (0.6 mL min^−1^). The detection was performed with a photo diode array (PDA) detector at 210 nm (Waters) and a refractive index (RI) detector (Waters 410) at 40 °C.

### Enzymatic assays

Liquid samples from the reactors were analyzed for cellobiohydrolase activity (CBH) as described previously (Xiros and Studer 2017), using crystalline cellulose (Avicel®, 2% *w*/*v*) as the substrate, at 50 °C for 1 h. All assays were performed in duplicate, in a thermomixer (Thermomixer® C, Eppendorf, Hamburg-Eppendorf, Germany) at 1400 rpm. The released sugars were measured with DNS reagent based on glucose as the standard sugar solution.

### Statistical analysis

The statistical significance of the differences observed among the results obtained from the different conditions tested was evaluated with analysis of variance (one-way ANOVA). The analysis was made with the software Sigmaplot 12.5. All fermentations were done in duplicate with 6-month difference between the two replicates. Detailed information on the schedule of the experiments is given in Table S1. In most cases, the measurements from both replicates are presented in the manuscript.

### Definitions of yields, selectivities

SCFA selectivity (g/g): individual acid produced/total SCFAs produced.

Total SCFAs yield (g/g): sum of acetic, propionic, butyric, and caproic acids/(initial substrate (cellulose)*1.1).

Carbon loss (g/g): carbon in the form of methane/carbon in the form of substrate.

## Results

All fermentation experiments were carried out in duplicate at different time points (half a year period between replicates). This way, all different parameters (pH, temperature, presence of fungal biofilm, methanogenic activity) were tested twice with potentially different starting rumen microbial communities. Although differences were observed in the absolute values of the results obtained between the replicates, the same trends regarding SCFAs yields, productivities, and selectivities were observed from the two duplicate sets for all conditions under investigation. Analysis of variance (one-way ANOVA) was performed with the raw values of the replicates to reveal potential differences. Although duplicate experiments were carried out at different times of the year, no statistically significant differences between the replicates were observed on SCFA production (Table 1). Similarly, no differences between the replicates were observed on the composition of the SCFA mixture.

**Table 1:** SCFA production using different amounts of rumen fluid volumes

Inoculum ratio	Acetic acid selectivity*		Butyric acid selectivity*		Total SCFA yield (g/g substrate)	
0.093	0.31	± 0.08	0.29	± 0.03	0.40	± 0.02
0.186	0.32	± 0.01	0.26 ^a^	± 0.06	0.39	± 0.03
0.279	0.30	± 0.06	0.33	± 0.01	0.39	± 0.01
0.372	0.35	± 0.01	0.40 ^a^	± 0.01	0.47	± 0.01

*SCFA selectivity is calculated as the ratio of the SCFA amount produced (g) to the amount of total SCFAs (g) produced at the end of fermentation. The presented values are the averages from two independent experiments. Significant differences among the different inoculum ratios for each category were evaluated with one-way ANOVA. A multiple comparison procedure (Tukeys) was also performed. The statistical different values for each column are marked with an ^a^

### Effect of the rumen inoculum size on SCFA production

The dry matter of the rumen fluid varied from 3.5 to 4.5% *w*/*v*, while the SCFAs content ranged from 12 up to 20 g/L. To transfer as less dry matter and SCFAs as possible in the reactors and also to have an efficient rumen inoculum for the in vitro bioconversion of cellulose to SCFAs, fermentation experiments were performed using rumen fluid/fermentation volume ratios from 0.093 up to 0.372. Although small differences were observed on the production patterns of SCFAs as a function of time (Fig. S1), no statistically significant differences were observed (Table 1) on the final SCFA yields and selectivities among the different ratios, apart from the butyric acid production between the ratios 0.186 and 0.372. With this being the only statistically significant difference, no trend could be observed regarding the production of total SCFAs, or that of butyric acid or any other acid, to justify the selection of a certain inoculum ratio.” Thus, the lower ratio tested (250 mL of rumen fluid in 2700 mL final volume, ratio 0.093) was applied in all later experiments with the rumen microbial community.

### The presence of a cellulolytic aerobic fungus in MBM reactors enhances the cellulolytic activities and the SCFA yields

To prove the suitability, the usefulness and the efficiency of the MBM system for SCFA production using the rumen culture, we investigated whether a fungal aerobic biofilm would be beneficial for the overall process, assuming that a higher cellulolytic efficiency would be reflected into higher SCFA productivities and yields.

**Fig. 1:**
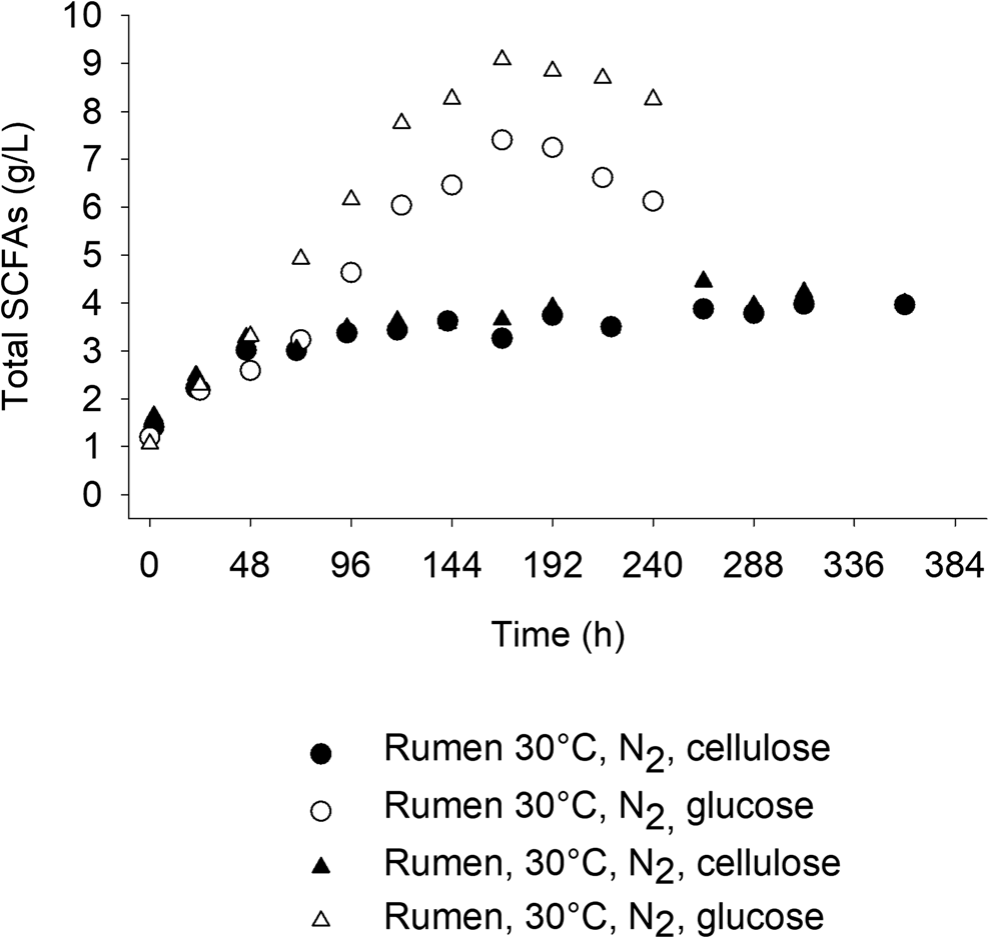
SCFA production by the rumen microbiome in the MBM system. Comparison of SCFA production using glucose (open shapes) or cellulose (dark shapes) as the substrate. The fermentations were carried out without a fungal biofilm on the membrane. The membrane was flushed with N_2_ during the experiment. The experiments were done in duplicate and both replicates are shown in the figure

To confirm the importance of the cellulolytic enzyme activities for an efficient conversion of cellulose to SCFAs by the rumen microbial community in the MBM system, experiments were carried out only with the rumen microbiome (no fungal biofilm present) using glucose as the substrate. The results from these experiments are compared with the results on cellulose conversion in Fig. 1. As shown, the rumen bacterial community converted glucose to SCFAs much more efficiently than cellulose, not only in terms of final yields but also in terms of productivities. It has been shown that neither crystallinity nor degree of polymerization is changing until enzymatic hydrolysis of Avicel reaches 50–60% conversion to sugars (Kafle et al. 2015). Taking also into account that Avicel is a quite homogeneous substrate and thus only a small increase to its recalcitrance during hydrolysis would be expected, it may be assumed that cellulose hydrolysis was a bottleneck of the process. This could be attributed to a progressive deactivation of the existing cellulolytic enzymes and a simultaneous modification of the composition of the rumen bacterial community, resulting in lower cellulolytic activities. More experiments were therefore designed to evaluate the ability of a fungal biofilm to enhance the cellulolytic activity during the conversion of cellulose to SCFAs by the rumen community. The temperature and pH optimum values for *Trichoderma reesei* and the rumen microbial community are very different and thus fermentations without *T. reesei* were carried out not only at pH 6 and T = 30 °C but also at 39 °C and pH 6.5 to eliminate any possible biases.

**Fig. 2:**
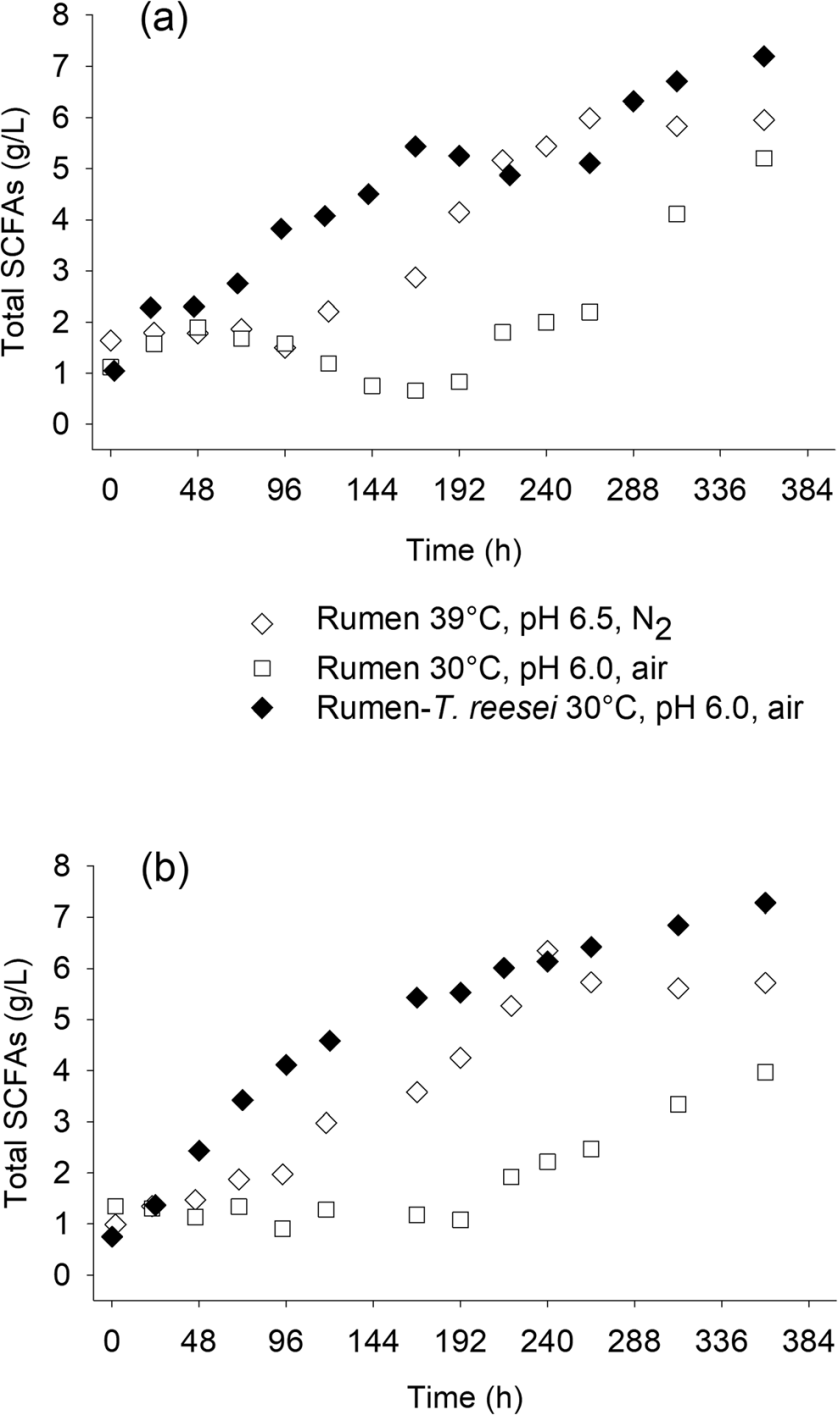
SCFA production during fermentations in the MBM reactors with or without *T. reesei* biofilm. Fermentations without *T. reesei* were performed at different pH and T values to enable the comparison at favorable conditions with 1.5% *w*/*v* crystalline cellulose as the cellulosic substrate. The experiments only with the rumen fluid were carried out with flushing air or nitrogen through the membrane. Time 0 is 48 h after the inoculation of *T. reesei*. To prevent erroneous results due to an increased methanogenic activity at 39 °C, pH 6.5, an inhibitor of the methanogens (2-bromoethane sulfonate) was added to prevent the carbon loss in the form of CH_4_. The fermentations were performed in duplicate (1st replicate shown in panel **a** and 2nd replicate shown in panel **b**) at different time periods (spring and fall) throughout the year

The results from fermentations carried out with or without a fungal (*T. reesei*) biofilm on the membrane of the reactor were compared. As shown in Fig. 2, at 30 °C, the presence of fungal biofilm resulted in higher SCFAs yield and productivity. At these conditions, the MBM system with *T. reesei* biofilm reached 7.3 g/L of total SCFAs, corresponding to a 39% higher concentration than the rumen microbiome alone (5.1 g/L). *T. reesei* biofilm also resulted in faster increase in SCFA concentration in the broth. Seventy-five percent of the maximum SCFA concentration had been reached after 168 h of cultivation, while in the case of the rumen microbiome alone (at 30 °C), SCFAs production was very low during the first 290 h of fermentation. When the membrane of the MBM system was not flushed with air but with N_2_, the lag phase was not observed. Therefore, it could be assumed that the lag phase was due to not strict anaerobic conditions in the reactor, during the early stages of the fermentation. This was supported by the redox profiles between these two cases (Fig. S2). However, with N_2_ flushing through the membrane, the SCFA yield from the rumen microbiome alone was even lower than in the case of air flushing.

Among the fermentations performed without a fungal biofilm, the more productive was at 39 °C and pH 6.5, conditions which are identical to the rumen natural conditions (Weimer et al. 2009) (Fig. 2). A lag phase (about 120 h), although shorter than the one at 30 °C, was again observed. At these conditions, the rumen microbial culture alone achieved a maximum concentration of 6.3 g/L (16% lower than the fermentation with *T. reesei*) from which 5.2 g/L is produced during the fermentation while 1.1 g/L was carried in the fermenter with the rumen fluid. Thus, the final SCFA concentration corresponded to a production yield of 0.32 g/g of cellulose, while when *T. reesei* was present, the production yield was 0.38 g/g of cellulose. The contribution of *T. reesei* in SCFA production and specifically to acetic acid production cannot be excluded since a paralog gene for aldehyde dehydrogenase (ALD1), which converts acetaldehyde to acetate, is not repressed by glucose (as it happens with the other paralog ALD2, and also with *Saccharomyces cerevisae*) (Chambergo et al. 2002). However, in previous experiments with *T. reesei* alone in the MBM system (Xiros and Studer 2017), acetic acid was not detected. Moreover, in all experiments, glucose and cellobiose were not detected in the medium throughout the cultivations indicating that cellulose hydrolysis was slower than sugar conversion to SCFAs.

**Fig. 3:**
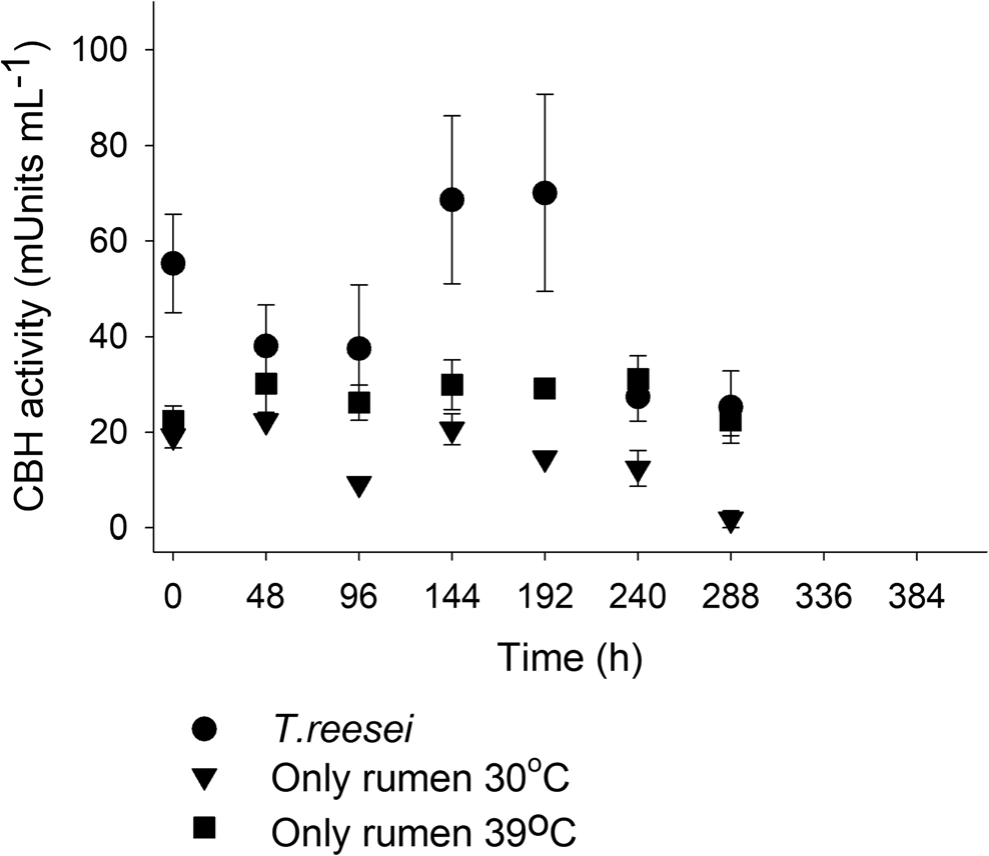
Effect of *T. reesei* biofilm on the CBH activity. Fermentations were carried out with 1.5% *w*/*v* of crystalline cellulose as the carbon source at pH 6 and at 30 °C (with or without *T. reesei* biofilm) or at 39 °C (only rumen inoculum). Error bars represent the range between minimum and maximum values between two independent fermentation experiments. All enzymatic assays were performed in duplicate and the mean values are shown

Measurements of free cellobiohydrolase activity (CBH) in the liquid medium gave an indication of the cellulolytic efficiency of the consortium, since CBH is the most representative enzymatic activity for the substrate (microcrystalline cellulose) used in the fermentations (Sharma et al. 2018). As shown in Fig. 3, at 30 °C, the presence of *T. reesei* resulted in higher CBH activities in the fermentation medium which is in accordance with the results on SCFA production shown in Fig. 2a, b. The CBH activities during the fermentations at 39 °C were slightly better than in 30 °C (for both fermentations in the absence of *T. reesei*), indicating that probably some cellulolytic microorganisms present in the rumen remain more active at higher temperature.

### Methanogenic activity during cellulose fermentations to SCFAs by the rumen microbial community

**Fig. 4:**
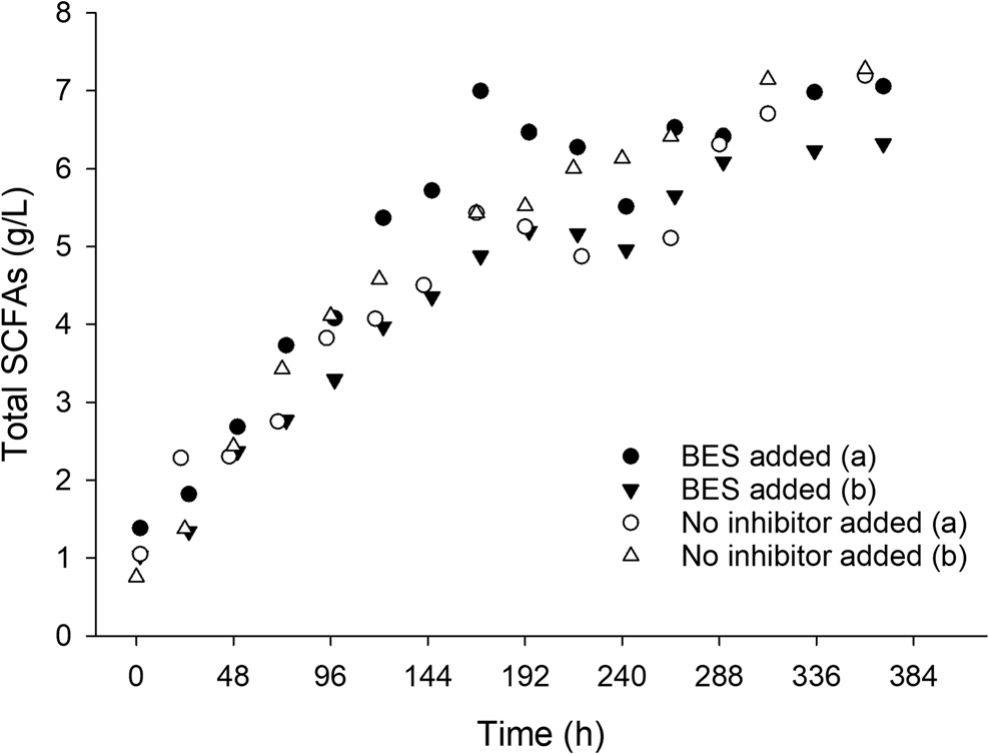
Effect of methanogenic activity on SCFA production at different process conditions. The methanogen inhibitor (BES) was added at a final concentration of 5 mM. Fermentations were carried out in the presence of a fungal biofilm (*T. reesei*) at pH 6, 30 °C. Duplicates were carried out at different time periods (spring and fall) throughout the year. Results from both replicates (**a**, **b**) are shown

Carbon loss to by-products should be minimized in order to maximize the yields of the conversion process. Methane was considered as a major potential by-product, since methanogenic microbial strains are part of the rumen microbiome (Moss et al. 2000). Therefore, the carbon loss in the form of methane was investigated and the effect of methanogenic inhibitors on the SCFAs yields was evaluated. The methane production during the experiments was measured with or without the addition of a chemical inhibitor of the methanogenic activity (Fig. S3). As shown, during the experiments with *T. reesei* biofilm, at 30 °C, without the methanogens’ inhibitor (2-bromoethane sulfonate), the accumulated methane production reached 0.4 g at the end of fermentation, while it was eliminated when the inhibitor was added. The initial carbon available in the form of cellulose was 16.2 g. The carbon loss (0.3 g) due to the CH_4_ production corresponded to 2% of the total carbon mass available in the form of cellulose, and thus was considered negligible. This is also reflected on the production of SCFAs with or without inhibition of the methanogens, where as shown in Fig. 4, no statistically significant difference was observed. Methane production during the fermentation with the rumen community alone was in all cases lower, even without the addition of a methanogens’ inhibitor, and even at 39 °C, which is the optimum temperature for rumen metabolism.

### Effect of pH on production and composition of SCFAs

**Fig. 5:**
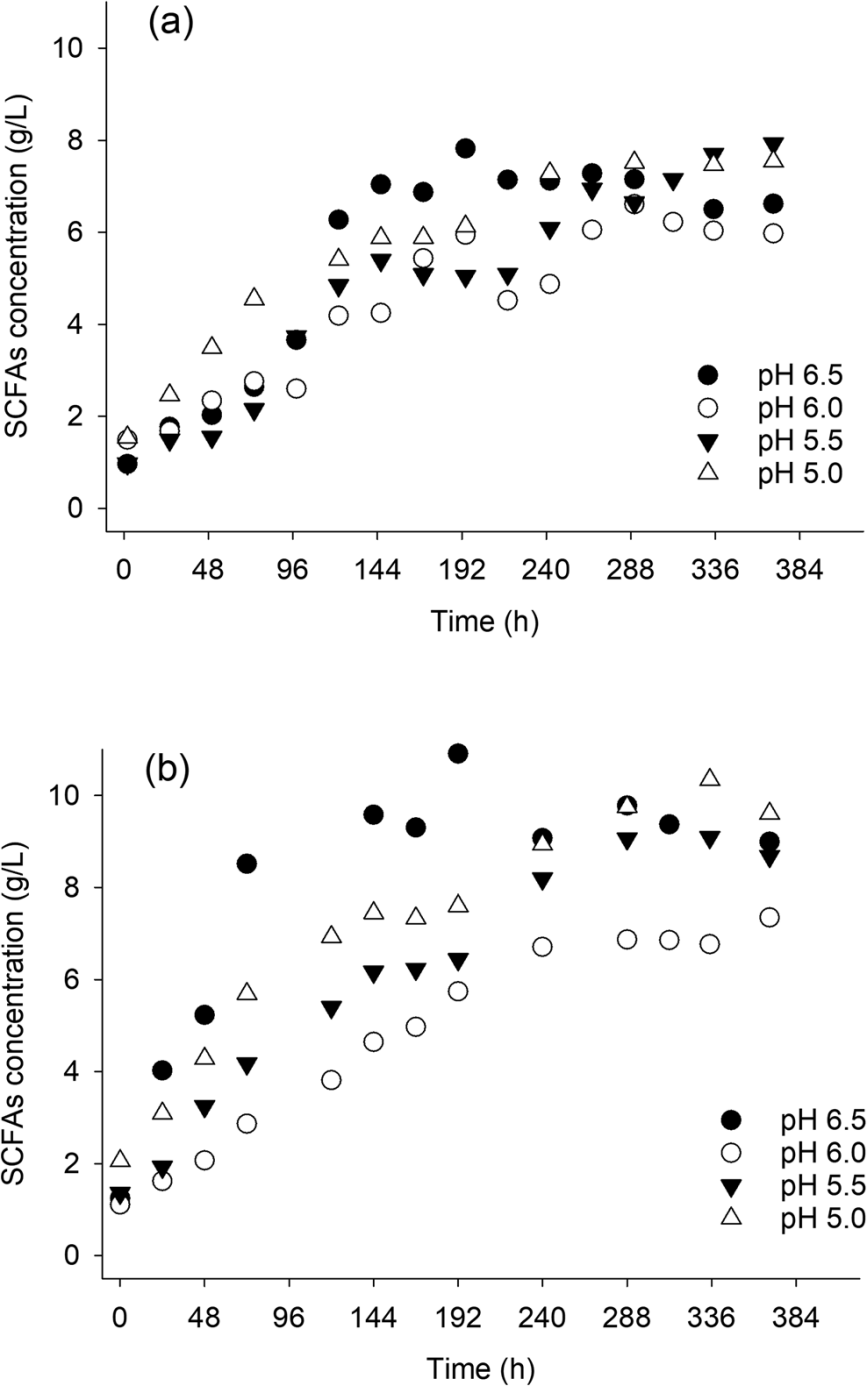
SCFA production at different fermentation pH values. Fermentations were carried out at a pH range from 5 to 6.5. All experiments were performed at 30 °C in duplicate, at different periods of the year: spring (**a**) and autumn (**b**). Crystalline cellulose (1.5% *w*/*v*) was used as the cellulosic substrate in all cases. The rumen fluid inoculum volume was 250 mL (ratio of rumen fluid to total volume 0.093)

The pH range to be studied was selected on the basis of the rumen physiological pH as well as the optimum pH of the fungus which formed the biofilm. Rumen pH ranges from 6.2 to 6.5 while the optimum pH for *Trichoderma reesei* is 5. The range that was therefore tested in the experiments with this fungus was from 5 to 6.5. In Fig. 5, the production of SCFAs for the different pH tested is shown. In the early stages of the fermentation, the acidogenic activity was higher at pH 5 and pH 6.5 than at pH 5.5 and pH 6. The highest SCFA yield (0.57 g/g of substrate) was achieved at pH 6.5 after 192 h of fermentation. At this point, the selectivities for acetic and butyric acid were 0.54 g/g and 0.22 g/g of total SCFAs, respectively (Fig. S4). However, at pH 6.5, the concentration of total SCFAs started to decrease after 200 h of fermentation. This decrease was not correlated to any methane production. The consumption of SCFAs could be attributed to consortium members which presumably are favored at higher pH values. At pH 5, a more stable production rate was maintained throughout the experiments probably also due to the better stability of the *T. reesei* cellulolytic enzymes at this pH resulting in a high cellulolytic efficiency for longer time.

Although duplicate experiments were performed at different time periods, and consequently they were inoculated with potentially different rumen microbial communities, the evolution of the SCFAs production did not change significantly from one period to the other (Fig. 5). However, an increase in all absolute values of SCFA concentrations can be observed in the case of the second set of replicates. These concentrations (presumably attributed to differences in inoculum) are also higher than those reported in previous experiments (Fig. 2, Fig. 4).

### Temperature strongly affects the composition of the produced SCFA mixtures

**Fig. 6:**
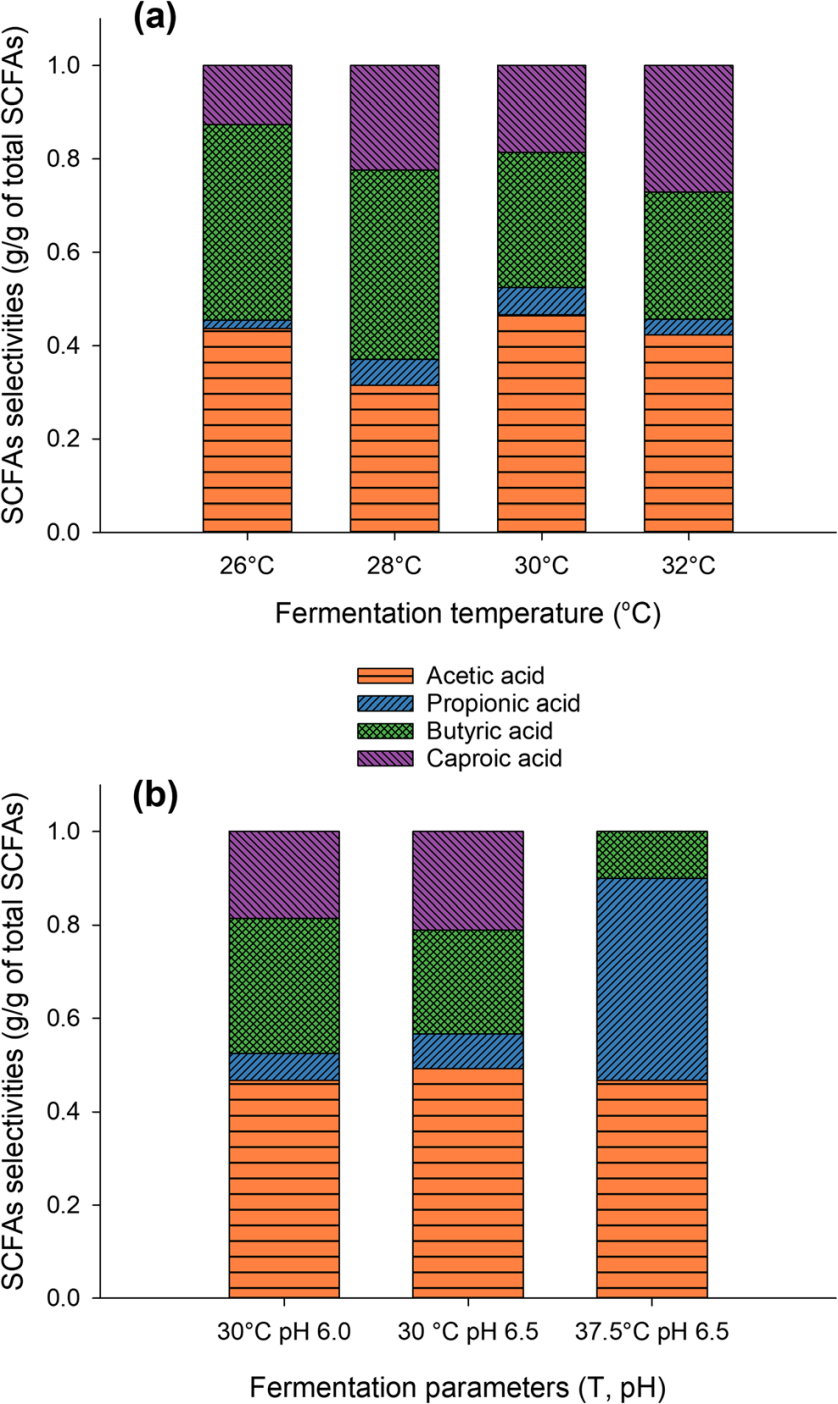
Effect of temperature on SCFA selectivities. **a** Fermentations at temperatures from 26 up to 32 °C with *T. reesei* as the fungal cellulolytic biofilm at pH 6. **b** Comparison of fermentation results obtained at 30 °C (with *T. reesei* at pH 6 and 6.5) and 37.5 °C, pH 6.5 (*C. cinerea*). All fermentations were carried out with 1.5% *w*/*v* crystalline cellulose as the cellulosic substrate. The results shown are the averages of fermentations performed in duplicate at different time periods throughout the year at the final sampling point except the fermentation at 37.5 °C with *C. cinerea* which was performed once

Fermentations were carried out at different temperatures to assess the effect of this parameter on SCFA production. Temperatures from 26 up to 32 °C were applied using *T. reesei*, while using *Coprinopsis cinerea*, fermentations were also performed at 37.5 °C. The fermentation temperature did not significantly influence the total SCFA production (online resource, Fig. S5). All experiments at temperatures from 26 to 37.5 °C gave very similar overall yields and productivities and only slightly higher overall yields were obtained from one replicate carried out at 32 °C. However, the temperature had a great impact on the composition of the SCFAs produced. As shown in Fig. 6a, acetic acid yield increased while butyric acid yield decreased. The ratio of produced acetic to butyric acid was 0.83 at 26 °C, while it reached 2.0 at 32 °C.

Experiments at 37.5 °C and pH 6.5 were carried out to study the SCFA composition at conditions closer to the optimum for the rumen microorganisms. *Coprinopsis cinerea* was used as the biofilm forming, cellulolytic fungus. Although a fermentation temperature of 39 °C would be also interesting to study, preliminary experiments showed that this fungal strain was growing better at 37.5 °C, and therefore, this temperature was selected. As shown in Fig. 6b, where the results of this experiment are compared with results obtained from fermentations at 30 °C (pH 6 and pH 6.5), this further increase of temperature had a dramatic effect on the composition of the SCFA mixture. Caproic acid was not detected in the production mixture throughout the fermentation and butyric acid concentration did not exceed 0.8 g/L, showing a decrease of about 400% compared with the results from lower temperatures. Acetic and propionic acids were practically the only acids produced, achieving selectivities (g of individual acid per g of total SCFAs produced) as high as 0.41 and 0.49, respectively. The acetic to butyric acid ratio was about 4.7 when the fermentations were stopped. The comparison of the three experiments in Fig. 6b indicated that although pH affected the acetic to butyric ratio to a certain extent (see also online resource, Fig. S4), the temperature had a higher impact on the SCFA composition, showing a clear trend from longer to shorter SCFAs when the temperature increased.

## Discussion

Numerous lab experiments studying the animal health and metabolism or the methane production have been carried out using the rumen microbial microbiome. In such *in vitro* experiments, the ratio of rumen fluid/fermentation volume is quite high (0.25 up to 1) (Agematu et al. 2017; Judd and Kohn 2018). However, if the rumen fluid is to be used for the hydrolysis of lignocellulosic substrates, not only the dry matter content of the rumen fluid, but also the SCFA content at those high ratios introduce complications and uncertainty on the efforts to calculate fermentation yields for the desired substrate.

In previous studies on the evaluation of the conversion of lignocellulosic substrates by the rumen microbial community, rumen fluid to final fermentation volume ratios from 0.1 to 0.2 has been reported (Weimer et al. 2011; Hernández-García et al. 2015; Murali et al. 2017). Therefore, having shown (Table 1 and Fig. S1) that the inoculum volume did not affect the overall SCFA production, and in line with previous studies, the ratio 0.093 has been chosen for the rest of the experiments in order to keep the solids and the SCFAs transferred to the reactors to the minimum possible amount.

The composition of the microbial community in the rumen is dependent on the diet of the animal (Deusch et al. 2017). Although the same procedure was followed for inoculum acquisition for all fermentations in this study, the changes in the cow diet throughout the year were considered as a source of variability affecting the composition of the rumen community and thus the cellulose bioconversion during the *in vitro* experiments.

### Role of fungal biofilm during cellulose bioconversion

Different kinds of mixed cultures and various carbon sources have been used for SCFA production. Aiello-Mazzari et al., using a version of the well-established MixAlco process, and employing a four-stage countercurrent system fermentation, achieved yields below 0.3 g /g (Aiello-Mazzarri et al. 2006) while other efforts with the MixAlco process achieved yields up to 0.55 g/g of total solids (Thanakoses et al. 2003a). The achieved SCFA overall yields (g of SCFAs per g of substrate used) during this study (~ 0.5 g/g) were similar to earlier results reported by Wang et al. (2014) using aerobic and anaerobic sludge inoculum (overall yields below 0.45 g of SCFAs per g of volatile solids). Wang et al. also calculated the SCFA yield on solubilized solids, estimating that about 90% of the solubilized solids were converted to SCFAs, indicating that hydrolysis was the bottleneck of that process, which is in agreement with many previous studies (Climent et al. 2007; Lee et al. 2014). All these reports suggest that enhancement of lignocellulolytic enzymatic activities would have a beneficial effect on the SCFA production.

Therefore, it is not surprising that the results obtained (Fig. 2 and Fig. 3) suggest that a *T. reesei* biofilm in the MBM reactor is beneficial for the SCFA yields and productivities if the rumen microbiome is to be used as the fermenting consortium at 30 °C. However, cellulolytic activities of many anaerobic bacterial species are organized in big ultrastructures (cellulosomes) attached to the substrate, fact that may have led to the underestimation of the cellulolytic activity of the rumen microbial community. Although the enzyme activities in the rumen are strongly dependent on the diet of the cow and especially on the ratio between grain and grass fed (Deusch et al. 2017), the changes in the cow diet during this study did not apparently result in very big differences in enzyme activities between the two experimental replicates.

Lower loss of carbon in the form of methane was observed during the fermentations without a fungal biofilm, compared to fermentations in the presence of a fungal biofilm (Fig. S3), showing that the fungal biofilm enhanced the metabolic activity of the rumen consortium in general. Although the differences in methane production are not very high, they show that the fungal biofilm was beneficial not only due to the enhanced cellulolytic activity of the system, but also due to the faster achievement of anaerobic conditions.

The complexity of the rumen makes a global and thorough description of the role of oxygen for the rumen metabolism very difficult. Although in the rumen, the great majority of microorganisms are strict anaerobic microbes, facultative anaerobic strains have also been found with unknown population frequencies and functionalities (Nagaraja 2016). The facultative anaerobes, as it has been shown earlier, can effectively decrease the redox potential resulting in anaerobic conditions even in case of membrane-based aeration in the MBM system (Shahab et al. 2018). However, the results obtained here showed that without the fungal biofilm, the decrease of the redox potential (probably due to the existence of these microorganisms) is very slow (online resource, Fig. S2).

### Methanogenic repression in the MBM system

In the natural environment of the methanogenic bacteria, the methane production varies depending on the cow diet and may reach from 250 L up to 500 L per day corresponding to an energy loss of 6–10% based on the energy content in the feed (Johnson and Johnson 1995; Immig 1996). However, not all the methane produced by cows is produced in the rumen. About 10–15% is produced by methanogenic populations in the intestine, so only 5–8.5% of the energy loss is due to the rumen methanogens. The energy loss observed during the experiments in the MBM reactors was lower. The accumulated methane produced was in all cases below 0.5 g, corresponding to an energy loss of less than 4% of the cellulose energy content. The small amount of methane produced during experiments in the presence of *T. reesei* may be partially explained by the experimental conditions (26–32 °C, pH 5–6.5) which were not optimum for the methanogenic populations (*Euryarchaeota*, *Bathyarchaeota*, *Verstraetearchaeota*), and partially, by the impossibility of withdrawing a representative sample of the whole rumen microbial community, since the cow rumen is quite inhomogeneous (distinct phases: liquid, solid, gas, and also various specific niches for various microbial species) (Hungate 1966; Weimer et al. 2009; Leng 2017) and it is therefore possible that a percentage of the methanogenic population was never carried in the reactors.

### Controlling SCFA selectivity in the MBM system

It is quite difficult to draw not only conclusions but even to make assumptions regarding the metabolic pathways prevailed during fermentations due to the complexity of the rumen consortium. The analysis of the microbial communities in the reactors would be necessary to this end, but even that would probably not lead to distinct conclusions. A very deep and accurate analysis would be needed, but even this, in the case of mixed communities would not guarantee the elucidation of the metabolic pathways (Deng et al. 2008; Deusch et al. 2017). However, assuming a strong selective pressure on the rumen microbial population at the experimental conditions, it could be probably assumed that much fewer microbial strains would prevail towards the later fermentation stage. Certainly, the environment in the MBM reactor is not at all identical to the rumen. This is indicated by the SCFA profiles at the end of the fermentations: in most cases, big differences (regarding the SCFA composition profiles) in the early stage of the experiments got smother towards the end of the experiments. Nonetheless, different pH values had an impact on the final SCFA composition. As shown in Fig. S4, longer SCFAs were produced in higher amounts at pH 5.5 and pH 6. The acetic acid selectivity was higher for pH 6.5 where the acidogenic activity resulted mostly in acetic acid production. This is in accordance with earlier studies on SCFA production using mixed undefined microbial cultures which showed increased acetic acid selectivity with pH increase (Dahiya et al. 2015). These individual SCFA selectivities achieved here for acetic and butyric acids are similar to the composition of SCFAs in the cow rumen and to those achieved earlier at similar fermentation temperatures (40 °C) (Thanakoses et al. 2003b).

The obtained results at different fermentation temperatures may be explained by two complementary reasonings. First, the increase in acetic acid selectivity as the temperature increased may be attributed to metabolism shifts of certain rumen microorganisms. The sugar catabolism of some acidogenic *Clostridia* species follows different pathways depending on the cells’ needs. At high growth rates, more acetic acid is produced while at lower ones, butyric production is relatively increased. It is the increased cell need of energy that drives the swift from butyric to acetic, since the pathway leading to acetic acid provides 4 ATP per g of glucose consumed, while that of butyric acid provides 3 ATP molecules to the cell (Girbal and Soucaille 1994; Dwidar et al. 2012). The effect of the ATP availability on acidogenic activity and selectivity is stronger in sugar-limited cultures (Girbal and Soucaille 1994) which was the case during this study where neither glucose nor cellobiose was detected in the broth during the experiments (due to the fact that hydrolysis rate is lower than fermentation rate). The temperature range 26 to 32 °C (suitable for *T. reesei*) was most probably suboptimum for the rumen microorganisms, and thus, they were growing in low growth rates favoring butyric acid production.

Second, temperature and pH are assumed to have a selective power on the rumen microbiome, and consequently, they affect the composition of the SCFA mixture produced, as certain groups of microorganisms in the rumen are more susceptible than others to temperature and pH changes. To confirm or reject these hypotheses, it is of course of great importance to identify and quantify the different microbial operational taxonomic units (OTUs) and compare them among the different conditions. To this end, the elucidation of the changes of the composition of microbial community in the MBM reactors during fermentations, as well as among the different conditions studied will be the next step of this investigation. The identification of crucial microbes regarding the metabolic shifts observed during this study will allow a better design and control of the process.

The rumen microbiome has been proved a promising fermentative consortium for the bioconversion of cellulosic substrates to SCFAs. The MBM reactors have been used successfully with different microorganisms and different applications (Brethauer and Studer 2014; Xiros and Studer 2017; Shahab et al. 2018). Scaling up of MBM reactors would require tackling the challenges regarding fungal biofilm formation, stability, and enzyme production and activity for a long period. Although the utilization of rumen fluid in large scale is not possible, the design of a synthetic consortium based on the community analysis of these experiments would be possibly promising for an industrial application. The enhancement of the cellulolytic activity of the consortium by the addition of selected fungi resulted in a faster fermentation, showing that cellulose hydrolysis was a bottleneck during the conversion. An economically viable application in commercial scale would be dependent on the viability and productivity of the fungal biofilm, which is something that must be further investigated. The results obtained during this study showed that the MBM system could be a versatile, selective system for SCFA production. The possibility to select and cultivate different fungi as the biofilm forming microorganisms offered the opportunity to investigate the effect of the process parameters on the fermentation characteristics. Temperature especially offers an effective tool to control the composition of the mixture of SCFAs produced. The possibility of different combinations of microorganisms at different process conditions is a powerful tool for a tuned, tailor made SCFA production according to desired applications and market needs.

## Electronic supplementary material

ESM 1 (PDF 892 kb)
