# Supplementary material for: A cellulolytic fungal biofilm enhances the consolidated bioconversion of cellulose to short chain fatty acids by the rumen microbiome
Source: Appl Microbiol Biotechnol. 2019 Mar 7;103(8):3355–65. doi: 10.1007/s00253-019-09706-1 (PMC6449290; doi:10.1007/s00253-019-09706-1)

Online resource file

Paper title:

A cellulolytic fungal biofilm enhances the consolidated bioconversion of cellulose to short chain fatty acids by the rumen microbiome

Journal: Applied Microbiology and Biotechnology

Authors:

Charilaos Xiros[1], Robert Lawrence Shahab[1,2], Michael Hans-Peter Studer [1*]

Affiliations

[1] Laboratory of Biofuels and Biochemicals, School of Agricultural, Forest and Food Sciences, Bern University of Applied Sciences (BFH), CH-3052 Zollikofen, Switzerland.

[2] Laboratory of Sustainable and Catalytic Processing, Institute of Chemical Sciences and Engineering, École Polytechnique Fédérale de Lausanne (EPFL), CH-1015 Lausanne, Switzerland

*Corresponding author: michael.studer1@bfh.ch

Table S1 Changes in the diet of the fistulized cow before and after calving

	Before calving			After calving			
	**ca.15.5.-21.5.2017**	**22.5.-28.05.2017**	**29.05.-7.6.2018**	**8.6.-18.6.2017**	**19.6.-25.6.2017**	**26.6.-2.7.2017**	**3.7.-9.7.2017**
Hay	ad lib	ad lib	ad lib	ad lib	ad lib	ad lib	ad lib
Maize cubes (whole plant) (kg/d)	0.5	1	1.5	1.5	2	2	2
Protein concentrate (kg/d)	0	0.25	0.5	0.5	1	1.5	1.75
Energy supplementation (Organic acids, vitamins, cereals, sugars) (kg/d)	0.5	1	1.5	1.5	2	2.5	3.5
**Mineral bolus**	Normal mineral bolus			Lactation mineral bolus		Normal mineral bolus	

Fig S1 Effect of rumen inoculum volume to final fermentation volume ratio on SCFAs production. Different rumen volumes were initially centrifuged, and the pellet was resuspended in 250 mL rumen fluid. Thus, in all cases the same volume of fluid was inserted in the reactor, but in each case, it contained a different number of rumen microorganisms corresponding to the initial rumen fluid volume. The fermentations were carried out in duplicate and both replicates are shown

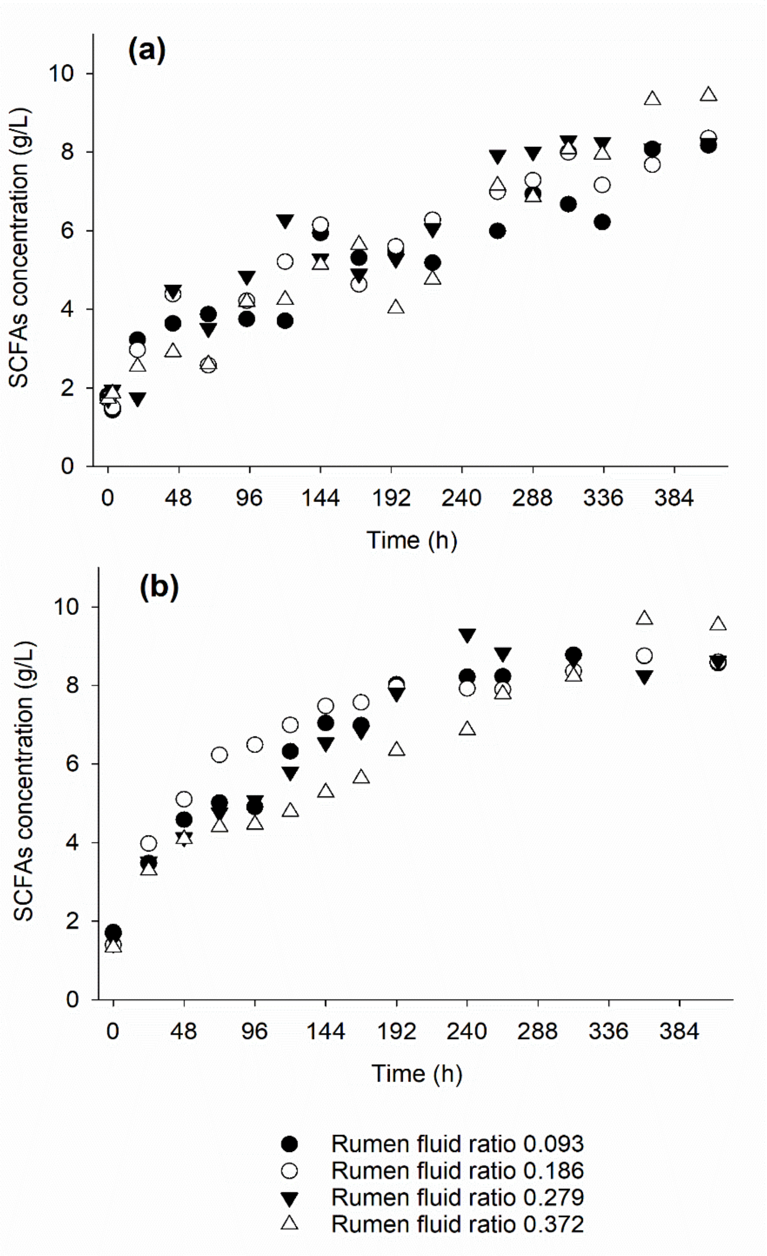

Fig S2 Evolution of the redox potential in the reactors, with or without the development of a fungal biofilm on the membrane. As zero, at the time scale, the moment of *T. reesei* inoculation is counted.

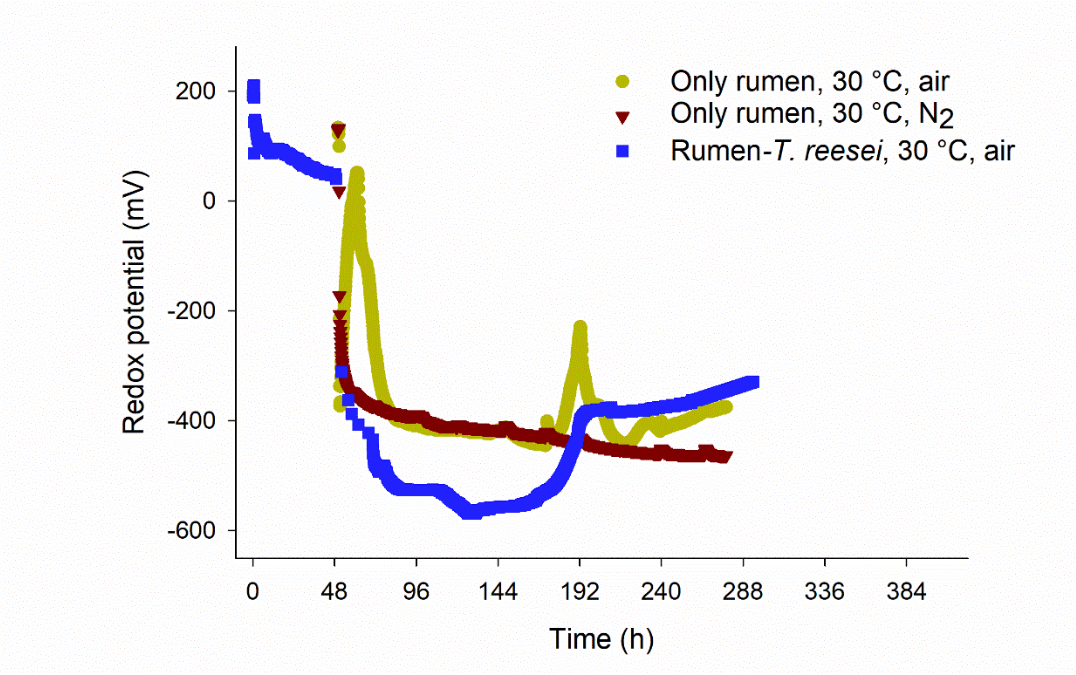

Fig S3 Methane production in the MBM system with or without the inhibition of the methanogenic activity. The accumulated amount produced is shown. BES concentration was 5 mM. The membrane was flushed with air during all co-cultivations with *T. reesei*.

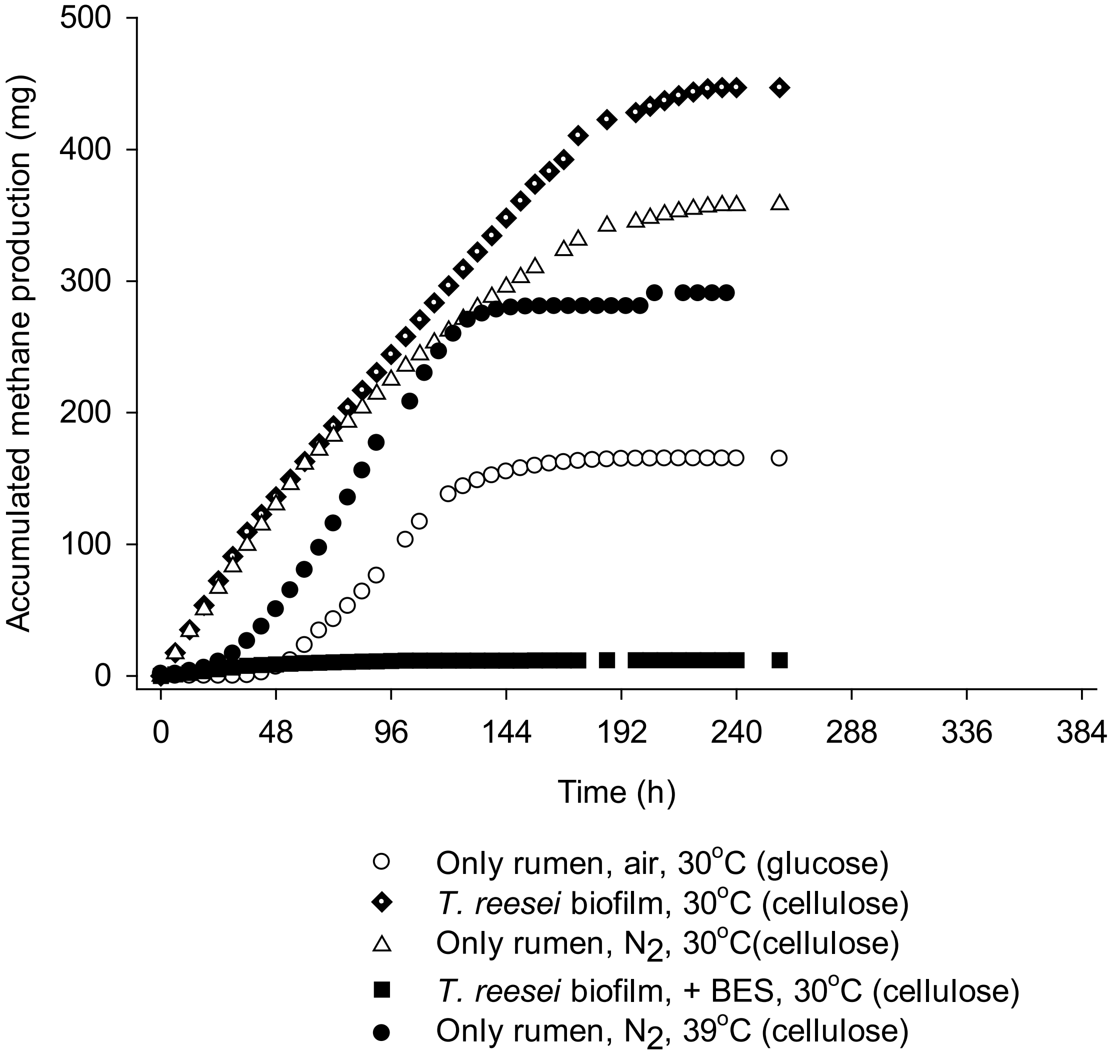

Fig S4 SCFAs selectivity in the MBM system at different fermentation pH values. The selectivity was defined as the ratio of the amount of a SCFA produced to the amount of total SCFAs (g/g).

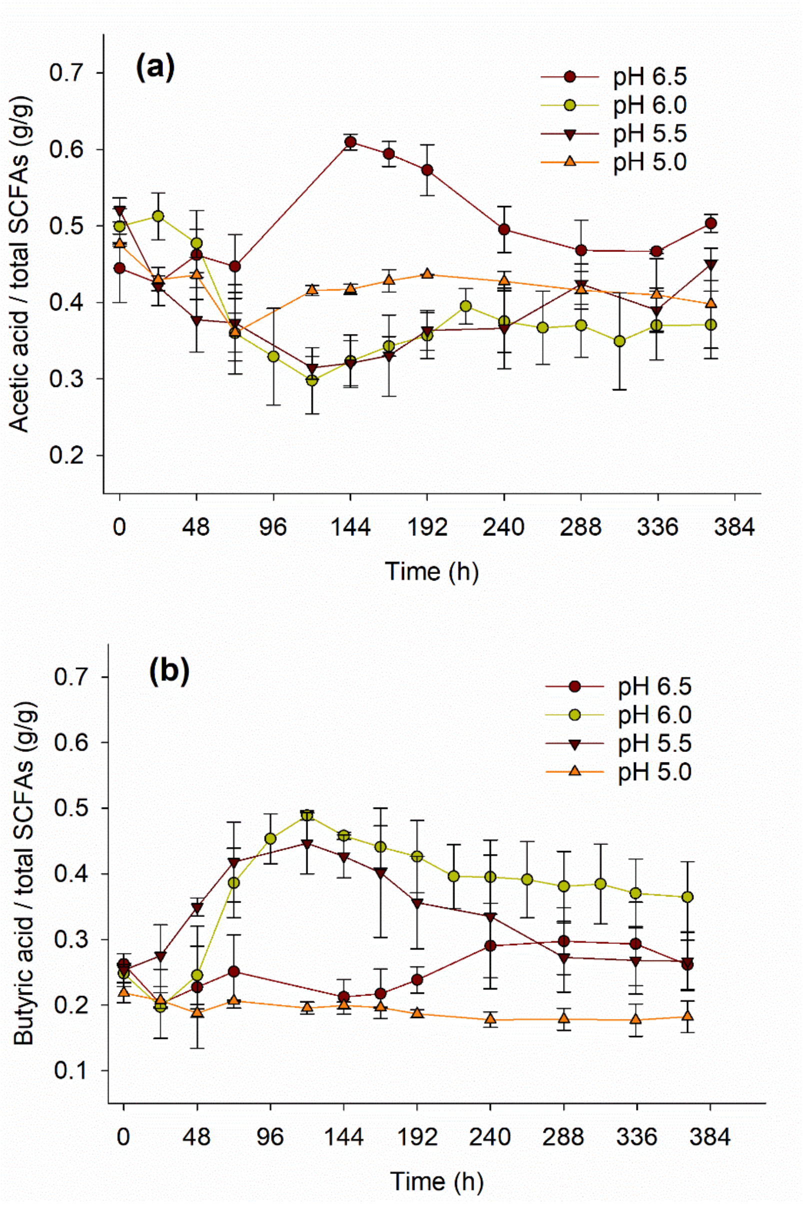

Fig S5 SCFAs production at different fermentation temperatures. Experiments with *T. reesei* (26°C – 32°C) were performed at pH 6 in duplicate, at different periods of the year: spring (A) and autumn (B). Crystalline cellulose (1.5 % w/v) was used as the cellulosic substrate in all cases. The experiment with *C. cinerea* was performed once during winter. The rumen fluid inoculum volume in all cases was 250 mL (ratio to total volume 0.093)

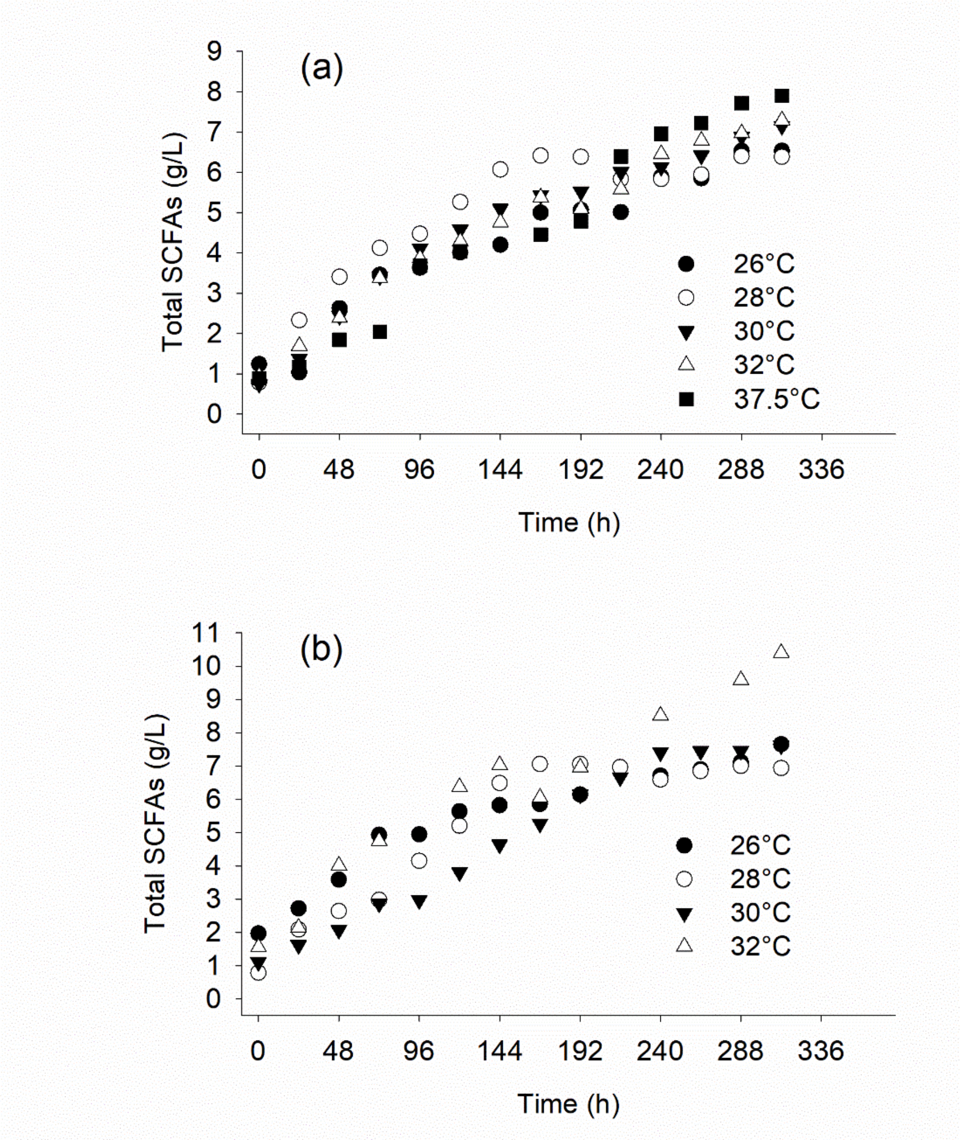

Supplement: Supplementary file 1 — (PDF 892 kb) [file 253_2019_9706_MOESM1_ESM.pdf]
